# Thiostrepton-Nanomedicine, a TLR9 Inhibitor, Attenuates Sepsis-Induced Inflammation in Mice

**DOI:** 10.1155/2023/4035516

**Published:** 2023-08-24

**Authors:** K. Esparza, S. D. Oliveira, M. Castellon, R. D. Minshall, H. Onyuksel

**Affiliations:** ^1^Department of Pharmaceutical Sciences, University of Illinois at Chicago, Chicago, USA; ^2^Department of Anesthesiology, University of Illinois at Chicago, Chicago, USA; ^3^Department of Physiology and Biophysics, University of Illinois at Chicago, Chicago, USA; ^4^Cardiovascular Research Core, University of Illinois at Chicago, Chicago, USA; ^5^Department of Pharmacology, University of Illinois at Chicago, Chicago, USA

## Abstract

Sepsis is a life-threatening clinical condition caused by infection and transposition of pathogens and pathogen-associated molecular patterns (PAMPs) into the host bloodstream. During sepsis, activation of toll-like receptors (TLRs) on immune cells triggers the release of pro-inflammatory cytokines and overstimulates the production of vasodilatory mediators such as nitric oxide (NO). These vascular changes lead to widespread inflammation, tissue damage, multiple organ failure, and often death. New therapeutic options are urgently needed. To this end, thiostrepton (TST) has emerged as a candidate for sepsis treatment due to its action as an antibiotic and anti-inflammatory molecule (TLR7-9 inhibitor). Reports in the literature suggest that TLR9 inhibition substantially suppresses the excessive host inflammatory response and attenuates sepsis-induced mortality in the cecal ligation and puncture (CLP) murine model of sepsis. However, to the best of our knowledge, TST has never been directly tested as a therapeutic option for the management of sepsis, possibly due to its low water solubility and drug delivery issues. These facts prompted us to test the central hypothesis that TST encapsulated in phospholipid sterically stabilized micelles (TST-SSM) could be developed into a novel treatment for sepsis. Thus, using our published method of encapsulating the hydrophobic antibiotic TST-SSM, we evaluated the *in vivo* efficacy of TST-SSM nanomedicine in the murine model of polymicrobial sepsis. We found that TST-SSM increased the median survival of CLP-induced septic mice from 31 to 44 hr by reducing the bacterial burden in the blood and peritoneal lavage. Moreover, plasma levels of pro-inflammatory cytokines (interleukin 6 and tumor necrosis factor-alpha) and NO derivatives were also reduced, whereas renal and hepatic function biomarkers creatinine and aspartate transferase were significantly improved. In conclusion, we identified that TST-SSM nanomedicine has significant potential as a therapeutic agent for sepsis management, primarily due to its anti-inflammatory and antibiotic properties.

## 1. Introduction

Sepsis is a complex life-threatening systemic syndrome caused by a dysregulated inflammatory response to an infection. Sepsis can occur as a result of different types of infection, including bacterial, viral, or fungal, which can evolve into persistent hypotension, hypoperfusion, multiorgan failure, and death [1]. Indeed, the Center for Disease Control (CDC) estimates that 1.7 million adults develop sepsis, with nearly 350,000 deaths yearly in the United States [2]. Although bacteria, viruses, and fungi can all cause sepsis and progress into septic shock, bacteria are the most common cause of infection. In line with those observations, a multicenter prospective study conducted with over 7,000 infected patients admitted to intensive care units across the globe, about 62.2% of microbial isolates were Gram-negative bacteria, 46.8% Gram-positive bacteria, and 19.4% fungi [3]. Moreover, the most common site of infections were the lungs (63.5%), abdominal organs (19.6%), bloodstream (15.1%), and urinary tract (14.3%), but other infected sites such as the skin and central nervous system can also lead to sepsis [3, 4].

The pathophysiology of sepsis is characterized by a dysregulated systemic immunological response against infection with significant life-threatening injury to the host [4]. Earlier definitions categorized sepsis into two phases: systemic inflammatory response syndrome (SIRS) and compensatory inflammatory response syndrome (CARS) [5]. In SIRS, the host innate immune system recognizes microbial patterns (pathogen-associated molecular patterns, PAMPs) via pathogen recognition receptors, including those of the toll-like receptor (TLR) family. PAMPs, in combination with products of injured/dying cells, trigger intracellular signaling cascades that promote the excessive secretion of pro-inflammatory cytokines and other mediators such as tumor necrosis factor-alpha (TNF-*α*), interferon-*γ* (IFN-*γ*), interleukin-1*β* (IL-1*β*), interleukin-8 (IL-8), interleukin-6 (IL-6), and reactive oxygen and nitrogen species. This “cytokine storm” prompts systemic inflammation by acting as endogenous pyrogens, activating the production of acute-phase proteins such as C-reactive protein (CRP), serum amyloid A (SAA), and fibrinogen are produced in the liver and play roles in the pathogen recognition, immune cell recruitment, and activation of the complement system. Also, in this phase, widespread endothelial activation occurs, promoting the expression of adhesion molecules and coagulation factors that contribute to leukocyte migration and promote vascular injury, thrombosis, and synthesis of secondary inflammatory mediators [6]. Thus, increased vascular permeability contributes to generalized edema, hypovolemia, compensatory tachycardia, and death. Given the severity of sepsis, early recognition and appropriate therapeutic interventions to reduce inflammation and bacterial burden are crucial to reducing sepsis-associated mortality [4].

During sepsis, the innate immune response via TLRs plays a central role in the inflammatory response. TLRs are highly expressed on the surface or intracellularly of various immune cells, including professional antigen-presenting cells such as dendritic cells and macrophages. Once TLRs recognize and bind to the PAMPs, they trigger a cascade of signaling events that activates other immune cells, which produce inflammatory cytokines, chemokines, and antibodies to resolve the infection [7]. TLRs are also involved in recognizing injured cells and initiating the tissue repair process. Among TLRs, intracellular TLR9 recognizes and binds to specific structures in the DNA of the pathogen, called CpG motifs, leading to immune cell activation and cytokine synthesis. TLR9 inhibition *in-vivo* by gene deletion [8, 9], small interfering RNA [10], and chemical inhibitors such as chloroquine [8] have been shown to significantly reduce the sepsis-related inflammatory response in animal models. Once activated, TLR9 triggers a downstream signaling cascade that results in the production of pro-inflammatory cytokines, including IL-1, IL-6, and TNF-*α*. These cytokines initiate and propagate the inflammatory response characteristic of sepsis. While the exact mechanism of TST-induced TLR9 inhibition is not entirely understood, research suggests that thiostrepton (TST) may bind directly to TLR9, preventing it from recognizing the CpG motifs inthe microbial DNA. Alternatively, TST could interfere with the downstream signaling pathways of TLR9, preventing the production of pro-inflammatory cytokines. Uncovering the details of the binding process, including the specific binding site and affinity, would be important for future research to further clarify the interaction between TST and TLR9 and to understand better the potential therapeutic role of TST in conditions characterized by the excessive TLR9 activation, such as sepsis. Therefore, inhibition of TLR9 activation represents a potential strategy for treating human sepsis.

In line with these observations, an antibiotic agent known as thiostrepton, or TST, has been reported as a potent TLR-9 inhibitor [11], but to the best of our knowledge, it has not been previously explored as a potential treatment for sepsis. TST is a cyclic oligopeptide naturally produced by several Streptomycetes strains, such as *Streptomyces azureus* and *Streptomyces laurentii*. It was first reported in 1955 [12], and since then, described as a potent bacteriostatic agent against Gram-positive bacteria such as Methicillin-resistant *Staphylococcus aureus* and Vancomycin-resistant *Enterococcus faecalis* [13]. TST is also described as anticancer [14], antiplasmodial, antimycobacterial [15], and anti-inflammatory activities [11, 16–18]. Despite being known for over half a century and has increased accessibility as a fully synthetic molecule, TST has never been developed into a commercial drug product for human use, possibly due to low water solubility and formulation challenges [13].

A safe and effective strategy to enable the use of hydrophobic drugs like TST is drug encapsulation in water-compatible vehicles such as phospholipid-based micelles, which can enhance the delivery of hydrophobic drugs like TST by improving their solubility, bioavailability, stability, and circulation time, and allowing for targeted delivery. In a previous publication by Esparza and Onyuksel [19], our group developed a novel nanomedicine of TST in sterically stabilized micelles (TST-SSM) using a scalable freeze–drying encapsulation process. These SSM are self-assembled nanosized structures (∼15 nm in diameter) of PEGylated phospholipids (DSPE-PEG_2000_) with a hydrophobic core and a hydrated hydrophilic shell. The hydrophobic core is able to accommodate hydrophobic drugs like TST in an active molecular form, while the hydrophilic shell protects the drug from elimination and enables circulation through the bloodstream. The solubility of TST increased from 3.5 to 833.4 *μ*M upon encapsulation in SSM (238-fold increase in drug solubility), and the nanomedicine still retained its potent *in vitro* antimicrobial activity [20]. The TST-SSM formulation also exhibited excellent *in vivo* anticancer activity, reducing tumor burden in mice inoculated with human high-grade serous ovarian cancer [14].

In this work, we report a novel application of TST nanomedicine as therapy for polymicrobial sepsis in the cecal ligation and puncture (CLP) mouse model. We enabled the intravenous delivery of TST by encapsulating this hydrophobic drug in safe and effective phospholipid-based SSM using a scalable method amenable to large-scale production. Our nanomedicine improved the survival time, reduced the bacterial burden, and improved various inflammatory markers in the septic mice, demonstrating its remarkable potential to treat human sepsis and reduce mortality.

## 2. Material and Methods

### 2.1. Animals

Wild-type male C56BL/6 mice were purchased from the Jackson Laboratory (Bar Harbor, ME) and maintained in standard cages in the UIC Biological Resources Laboratory (BRL). Animals were allowed at least 48 hr for acclimatization before use. Five mice were housed in each cage under a 12 hr light/dark cycle. Animals were fed a normal chow diet and drinking water *ad libitum*. All animal study protocols were approved by the Animal Care Committee (ACC# 17-138 Mod 2) at UIC.

### 2.2. Preparation and Characterization of TST-SSM Nanomedicine for In Vivo Experiments

TST-SSM nanomedicine was prepared by the cosolvent freeze-drying method and characterized regarding its visual appearance, particle size, and drug concentration. The TST-SSM formulation and production process has been fully described in a previous publication [19]. In summary, stock solutions of TST (555.4 *μ*M) and DSPE-PEG_2000_ (10 mM) were prepared in *t*-butanol and 0.667x phosphate-buffered saline, respectively. The DSPE-PEG_2000_ dispersion was slowly added to the TST solution under constant stirring at a 50 : 50 proportion. The solution was transferred to glass vials (6 mL in each) and froze overnight at −80°C. The frozen material was freeze-dried for 24 hr using a Labconco Freeze Dry System FreeZone® 4.5 floor model catalog number 77510–00 (Labconco, Kansas, MO) using default parameters (≤0.133 mbar of vacuum pressure and condenser temperature at ≤−40°C). Freeze–dried material was kept at −80°C until use. On the day of the experiment, samples were reconstituted with sterile ultrapure water (2 mL per vial) and gently swPirled to achieve a ∼15 nm micellar formulation with a final concentration of 833 *μ*M of TST and 15 mM of DSPE-PEG_2000_ in 1x phosphate buffer saline (PBS). Empty SSM (control) was prepared on the day of the experiment by dissolving DSPE-PEG_2000_ in sterile 1x PBS to a final concentration of 15 mM.

### 2.3. Induction of Polymicrobial Sepsis in Mice by the Cecal Ligation and Puncture (CLP) Method

**Figure 1:**
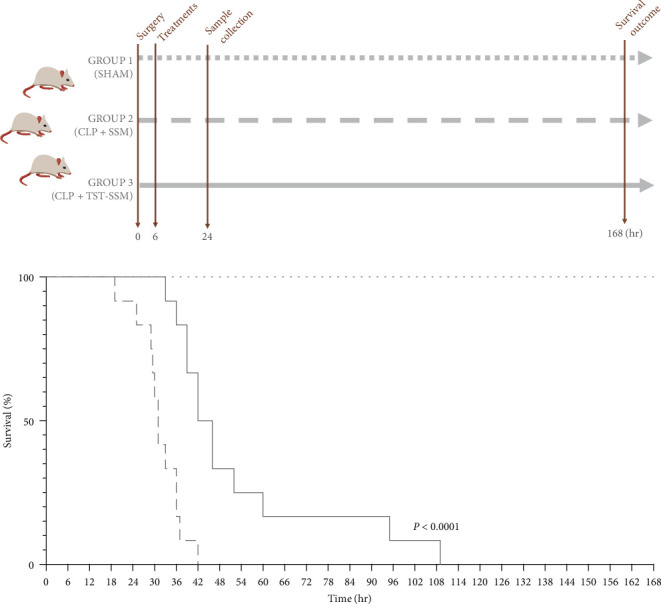
Experimental groups and survival study of mice with CLP-induced sepsis. (a, b) C57BL/6 mice (12 per group) underwent cecal ligation and puncture (CLP) or SHAM surgery (time 0). At 6 hr, CLP mice were either treated with empty micelles (SSM) or 20 mg/kg thiostrepton nanomedicine (TST-SSM) intraperitoneally and monitored for survival up to 7 days (168 hr). Survival rates were compared using the log-rank (Mantel-cox) test. *P* < 0.05 was considered statistically significant.

The CLP model from a previously published method by Herrmann et al. [21] was used to evaluate the therapeutic value of TST-SSM nanomedicine for treating polymicrobial sepsis in mice. The study was divided into two parts to assess survival and physiological status (Figure 1(a)). In brief, animals were anesthetized with an intraperitoneal (i.p.) injection of ketamine (100 mg/kg) and xylazine (10 mg/kg). Upon reaching a surgical plane of anesthesia, the abdomen was carefully shaved and rubbed with povidone–iodine antiseptic solution before making a 1 cm skin incision on the left side. The underlying abdominal muscle was incised with scissors, exposing the cecum. The distal 20% of the cecum was ligated with a 6-0 suture between the first and the second groove from the tip to interrupt blood circulation. Through and through punctures were made once in the ligated area and twice in the nonligated area with a 20 G needle. The cecum was lightly pressed to extrude a defined amount (0.5 cm^2^) of intestinal content and placed back in the abdominal cavity. The peritoneal cavity was closed in two layers (muscle and skin) with 6-0 sutures. SHAM animals underwent the same surgical procedure (anesthesia, abdominal incision, suture, pre- and postsurgical care) but without ligation and puncture of the cecum. All mice received 0.5 mL of warmed PBS via subcutaneous injection on the left flank after surgery as support therapy. The CLP procedure was performed within 1.5 hr to reduce variations in the experimental results. Animals presenting with sepsis were treated 6 hr after the CLP surgery via i.p. injection (max. volume 0.5 mL) with empty micelles (negative control) or 20 mg/kg TST-SSM (treatment). This time point of treatment was selected to allow time to establish systemic inflammation after the CLP procedure [22]. The TST dose was fixed at 20 mg/kg, below the reported IV LD50 of 41 mg/kg (“Toxnet-TST USP,” n.d.). Animals were monitored every 6 hr for the first 24 hr and twice daily afterward. For the survival study, animals were monitored for 7 days. Euthanasia was performed by isoflurane overdose and cervical dislocation once animals achieved humane endpoints (labored breathing and lack of purposeful movement) or at the end of the study. For the physiological status study, animals were euthanized 24 hr after CLP surgery. To collect the blood, we injected 50 *μ*L of heparin (1,000 units/mL) into the inferior vena cava and allowed it to circulate for a minute before drawing the anticoagulated blood. We immediately transferred 10 *μ*L of blood into a sterile Eppendorf tube containing 90 *μ*L of 1x PBS in triplicate for the bacterial count. The remaining blood was centrifuged at 3000 × *g* for 15 min. Plasma samples were analyzed immediately or stored at −80°C until assayed. Finally, 1 mL of sterile 0.9% saline was instilled into the peritoneal cavity and massaged the abdomen gently for a few seconds. The peritoneal lavage (∼0.5 mL) was recovered to determine the bacterial count [23].

### 2.4. Enzyme-Linked Immunosorbent Assay (ELISA)

Pro-inflammatory (IL-6 and TNF-*α*) and anti-inflammatory (IL-10 and TGF-1*β*) cytokines were quantified in plasma samples using ELISA kits from RnD Systems following the manufacturer's protocols.

### 2.5. Measurement of Nitric Oxide Derivatives (NO_x_) in Plasma by Griess Assay

The total nitrate/nitrite (NO_*x*_) was determined spectrophotometrically in plasma samples with the Griess Reagent Kit as an index of nitric oxide (NO) level. NO is a labile molecule rapidly converted into nitrite (NO_2_−) and nitrate (NO_3_−) in an aqueous solution. Since the Griess reagent detects nitrite only, we converted nitrate to nitrite first using NADH-dependent enzyme nitrate reductase. We mixed 80 *μ*L of the sample, 10 *μ*L of the cofactor preparation, and 10 *μ*L of nitrate reductase, followed by 3 hr incubation at room temperature. The Griess reaction was performed according to the manufacturer's protocol.

### 2.6. Blood Chemistry

Biomarkers of renal function (blood urea nitrogen (BUN) and serum creatinine) and hepatic function (alanine transaminase (ALT), and aspartate transaminase (ASP)) were analyzed in fresh plasma samples using a Beckman Colter AU480 Chemistry Analyzer (Brea, CA) in the UIC Biological Resources Laboratory.

### 2.7. Bacterial Burden

The bacterial burden was assessed in fresh peritoneal lavage fluid and blood. Peritoneal lavage fluid was serially diluted tenfold in sterile 1x PBS and 10 *μ*L of each diluted sample was plated on tryptic soy agar. The plates were incubated at 37°C for 18–24 hr when the bacterial load was quantified by counting colony forming units (CFUs) on each plate [23]. The bacterial load was calculated using the formula

(1) $$\begin{array}{c} \text{bacterial load}\left(\mathrm{CFU}/\mathrm{mL}\right)=\left(\text{number of CFUs on plate}\times 10^{3}\right)\times \text{dilution}. \end{array}$$

### 2.8. Reagents and Materials

1,2-Diastearoyl-sn-glycero-3-phosphoethanolamine-N-methoxy-poly(ethyleneglycol 2000) (DSPE-PEG_2000_) sodium salt was purchased from LIPOID GmbH (Ludwigshafen, Germany). TST (purity ≥98%) was purchased from EMD Millipore (Burlington, MA). Molecular biology grade water and PBS 1x were obtained from Corning (Corning, NY). Sterile 0.9% saline was purchased from Baxter (Deerfield, IL). Water and 0.9% sodium chloride for injection were from Hospira/Pfizer (Lake Forest, IL). Tertbutanol (TBA), and tryptic soy agar, were purchased from Sigma–Aldrich (St Louis, MO). Scintillation glass vials of 4 and 20 mL were obtained from Kimble-Chase (Vineland, NJ). Serum tube glass vials (20 mL), rubber stoppers, and flip-top caps were purchased from Fisher Scientific (Waltham, MA). Millex GP polyethersulfone (PES) sterile syringe filters (0.22 *μ*m) were acquired from Millipore (Burlington, MA). IL-6, IL-10, TNF-*α*, and TGF-*β* ELISA kits were purchased from R&D Systems (Minneapolis, MN). The Griess Reagent Kit was obtained from Thermo Fisher Scientific (Waltham, MA). Nitrate reductase and nitrate reductase cofactor preparations were obtained from Cayman Chemical (Ann Arbor, MI). Isoflurane was obtained from Piramal Critical Care (Telangana, India). Lidocaine hydrochloride (20 mg/mL) was obtained from APP Pharmaceuticals (Schaumburg, IL). Ketamine hydrochloride injection was obtained from West-Ward (Eatontown, NJ). Xylazine injection was obtained from Akorn (Lake Forest, IL). Heparin sodium injection (1,000 USP units/mL) was from Aurobindo Pharma Limited (Pashamylaram, India).

### 2.9. Statistical Analysis

Data are presented as the arithmetic mean +/− standard error of the mean. Shapiro–Wilk test was used to determine the normality of data, and the Brown–Forsythe or F test was used to assess the equality of variances. Then, a parametric or nonparametric test was performed accordingly. Normally distributed data with two groups were compared using a Student's *t*-test. One-way ANOVA analyzed studies with more than two groups and one independent variable with Tukey's multiple comparisons tests. Two-way ANOVA analyzed studies with more than two groups and two independent variables with Tukey's multiple comparisons test. Survival curves were created using the Kaplan–Meier method, and differences were compared with the log-rank (Mantel-Cox) test. Bacterial burden results in blood and peritoneal lavage were log-transformed and analyzed by a one-tailed Mann–Whitney test (nonparametric). GraphPad Prism 7.01 and 8.0 software (San Diego, CA) were used for all statistical analyses. A *p*-value < 0.05 was considered statistically significant.

## 3. Results

### 3.1. TST-SSM Nanomedicine Improved Survival in the Murine Model of CLP-Induced Sepsis

The *in-vivo* efficacy of TST-SSM nanomedicine was evaluated by treating CLP-induced septic mice with either TST-SSM (treatment group) or empty SSM (negative treatment control). A SHAM-operated group (nonseptic) was included to minimize the surgical stress or operative care as a confounding factor in survival rates or inflammatory markers (Figure 1(a)). Our data indicated that a single i.p. dose of TST-SSM nanomedicine (20 mg/kg) administered 6 hr after CLP surgery extended the median survival time of septic animals from 31 to 44 hr (Figure 1(b)). All septic animals treated with empty micelles (control) perished within 2 days of the experiment versus 5 days for the group who received TST-SSM nanomedicine. All SHAM-operated animals survived until the end of the experiment (7 days).

### 3.2. TST-SSM Nanomedicine Reduced Pro-Inflammatory Markers in the Murine Model of CLP-Induced Sepsis

**Figure 2:**
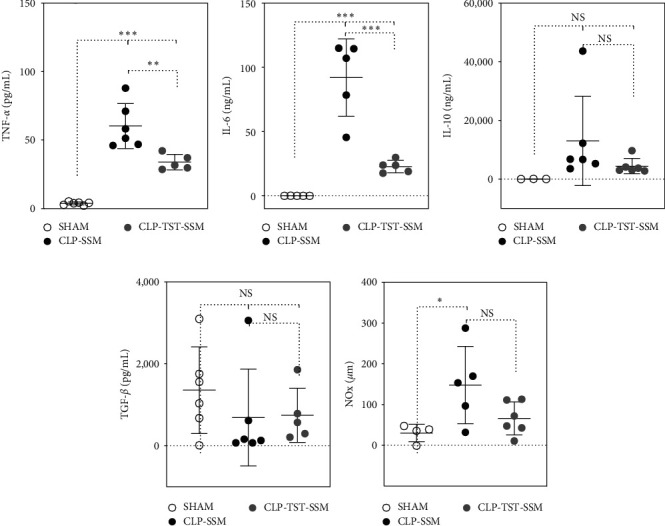
Cytokine profile and nitric oxide derivatives (NO_*x*_) levels in the plasma of mice with CLP-induced sepsis. C57BL/6 mice (3–6 per group) underwent cecal ligation and puncture (CLP) or SHAM surgery. CLP animals received empty micelles (SSM) or thiostrepton nanomedicine (TST-SSM) intraperitoneally at 6 hr, and all animals were euthanized at 24 hr. TNF-*α* (a), IL-6 (b), IL-10 (c), TGF-*β* (d), and nitrite/nitrate levels (NO_*x*_) (e) were quantified from heparinized plasma samples using ELISA kits (cytokines) and Griess Reaction Kit (NO_*x*_). Values are mean ± standard deviation. One-way ANOVA with Tukey's multiple comparison test was used to analyze the results. *P* < 0.05 was considered statistically significant. NS, not significant,  ^*∗*^*P* < 0.05,  ^*∗∗∗*^*P* < 0.001, and  ^*∗∗∗∗*^*P* < 0.0001.

A single i.p. dose of TST-SSM reduced systemic pro-inflammatory markers IL-6 and TNF-*α* compared to empty micelles (Figures 2(a) and 2(b)). On the other hand, the levels of anti-inflammatory cytokines IL-10 and TGF-*β* showed no statistical difference among CLP-induced septic mice (treatment and control) and SHAM controls (Figures 2(c) and 2(d)). Septic mice treated with empty micelles had significantly higher levels of NO_*x*_ than nonseptic SHAM controls, but no significant differences were observed between the treatment and treatment control groups (Figure 2(e)).

### 3.3. TST-SSM Significantly Reduced Plasma Creatinine but Failed to Improve BUN Levels

**Figure 3:**
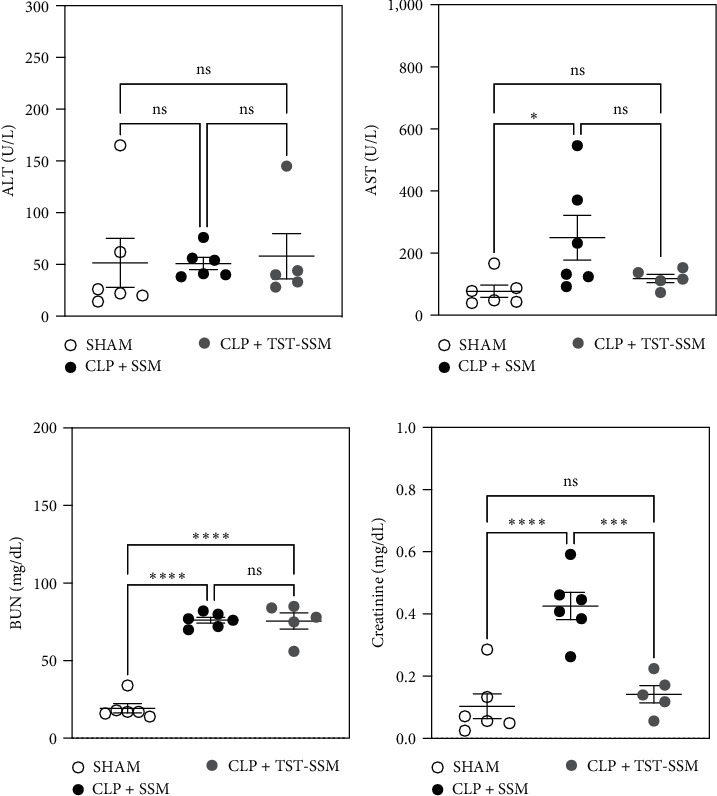
Biomarkers of hepatic and renal injury. C57BL/6 mice (5–6 per group) underwent CLP or SHAM surgery. CLP animals received empty micelles (SSM) or thiostrepton nanomedicine (TST-SSM) i.p. at 6 hr, and all animals were euthanized at 24 hr. (a, b) Hepatic biomarkers (AST and ALT) and (c, d) renal biomarkers (BUN and creatinine) were measured from heparinized plasma samples. Values are mean ± standard error mean. One-way ANOVA analyzed results with Tukey's multiple comparisons. Ns, nonsignificant; AST, aspartate transferase,  ^*∗*^*P* < 0.05,  ^*∗∗∗*^*P* < 0.001, and  ^*∗∗∗∗*^*P* < 0.0001.

Plasma levels of classical hepatic and renal function biomarkers were compared among SHAM and CLP animals euthanized at 24 hr. The purpose was to determine if TST-SSM treatment reduces organ damage commonly observed in sepsis. The hepatic marker ALT was unchanged among all groups (Figure 3(a)). Aspartate transferase (AST) was significantly higher in septic mice treated with empty micelles than in SHAM-control, but no statistical differences were observed in animals treated with TST-SSM (Figure 3(b)). Regarding the renal biomarkers, we observed mixed results. TST-SSM failed to reduce or prevent the increase of BUN levels (Figure 3(c)), but it significantly reduced plasma creatinine levels compared to animals treated with empty micelles (Figure 3(d)). Since plasma creatinine is a more specific indicator of renal function than BUN, its significant reduction with TST-SSM treatment, despite the lack of improvement in BUN levels, suggests a potential nephroprotective effect.

### 3.4. TST-SSM Reduced Intravascular and Peritoneal Bacterial Burden in the Murine Model of CLP-Induced Sepsis

**Figure 4:**
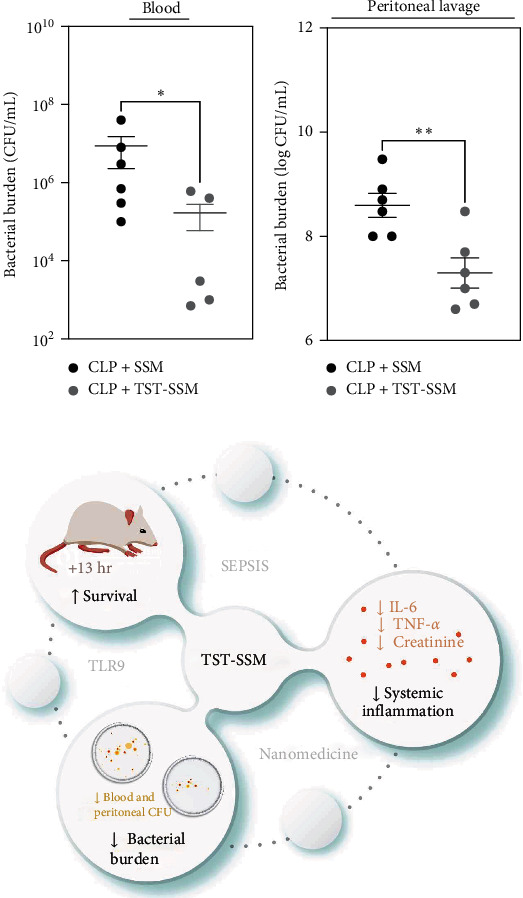
Bacterial burden in blood and peritoneal lavage fluid. C57BL/6 mice (5–6 per group) were subjected to CLP surgery or SHAM surgery and received empty micelles (SSM) or thiostrepton nanomedicine (TST-SSM) via intraperitoneal injection at 6 hr after surgery. All animals were euthanized at 24 hr, and fresh blood (a) and peritoneal lavage fluid (b) were collected for microbiological culture. Results were log-transformed and analyzed by a one-tailed Mann–Whitney test. *P* < 0.05 was considered statistically significant.  ^*∗*^*P* < 0.05,  ^*∗∗*^*P* < 0.01. (c) Summary of research results. CFU, colony forming units; TST-SSM, TST nanomedicine; IL-6, interleukin 6; TNF-*α*, tumor necrosis factor-alpha; TLR9, toll-like receptor 9; hrs, hours.

Finally, to determine whether TST-SSM treatment reduced the microbial burden in septic mice, we evaluated the bacterial counts in fresh blood and peritoneal lavage fluid of SHAM and CLP animals. At 24 hr after CLP, we observed that TST-SSM treatment significantly reduced the bacterial burden in both blood (Figure 4(a)) and peritoneal lavage (Figure 4(b)). No bacterial growth was observed in SHAM-control animals. The observed reduction in bacterial burden following TST-SSM treatment suggests that, in addition to its immunomodulatory effects, TST-SSM preserves TST antimicrobial properties. These could be attributed to the TST-SSM altering the host environment, making it less hospitable for bacterial growth or directly inhibiting bacterial proliferation or survival.

## 4. Discussion

TST is a potent TLR9 inhibitor able to reduce the excessive skin inflammation in a psoriasis mouse model [11]. Moreover, TLR9 inhibition by gene deletion [8, 9], small interfering RNA [10], and chemical inhibitor (chloroquine) [8] has been shown to reduce the excessive inflammation and mortality of CLP-induced sepsis, a clinically relevant model of polymicrobial sepsis. Despite this connection, the use of TST for the management of sepsis remains unknown, probably due to its low water solubility and formulation challenges. Therefore, in this work, we tested for the first time whether TST encapsulated in phospholipid micelles (TST-SSM) attenuated sepsis in a murine model of CLP-induced polymicrobial sepsis.

Previous studies have demonstrated the effect of TLR9 inhibition on sepsis survival [9, 24, 25]. Hu et al. [9] observed a survival rate of 70% in TLR9 knockout Balb/c mice after CLP surgery compared to 20% in the wild-type mice. Similarly, Liu et al. [10] observed that after silencing TLR9 expression in C57Bl/6 mice with TLR9 siRNA plasmid, treated animals exhibited 80% survival compared to approximately 20% in the untreated group [9, 10]. Therapeutic approaches have also demonstrated a positive effect on improving survival. For example, a 24% versus 55% survival rate of 32–40 weeks old C57Bl/6 mice was observed upon chloroquine treatment versus animals treated with vehicle-only following CLP surgery [8]. In this case, CLP surgery was performed with two punctures, and animals received broad-spectrum antibiotics as a supportive measure which likely contributed to the outcome. In line with these observations, administering a higher dose or multiple doses of TST-SSM could lead to an even better survival benefit in treated animals. Moreover, additional therapeutic strategies, such as combining TST-SSM nanomedicine with conventional broad-spectrum antibiotics, could synergistically improve survival and accelerate the time for recovery of animals with severe sepsis.

Sepsis represents a complex interplay between the body's pro-inflammatory response, which is necessary to combat infection, and excessive inflammation that can lead to tissue damage and organ dysfunction. The modulation of cytokine levels, as seen with TST-SSM treatment, could help to restore this balance. The timing of our study, capturing the acute phase of sepsis, is particularly pertinent as it is during this phase that immune dysregulation is most severe, and treatment interventions could have the most impact. In line with these observations, besides improving survival in the murine model of CLP-induced sepsis, TST-SSM also demonstrated an important effect in dampening circulating pro-inflammatory mediators, such as IL-6 and TNF-*α*, compared to SHAM animals. Pro-inflammatory cytokines, such as IL-6 and TNF-*α*, help to initiate the inflammatory response necessary to control infection and prevent its systemic spread [6, 7]. However, excessive pro-inflammation also promotes widespread tissue damage and organ dysfunction, which can culminate in septic shock [26, 27]. Cytokines also play a role in the later stages of sepsis, contributing to the development of immunosuppression. In this stage, cytokines such as IL-10 and TGF-*β* are released, suppressing the immune response and making patients and animal models even more susceptible to the secondary infections [28]. Here, our data showed no differences in the anti-inflammatory cytokines IL-10 and TGF-*β* among SHAM and CLP animals after 24 hr from the surgery. This suggests that animals were still in the acute phase of sepsis, and no differences in the late-phase cytokines could be captured in our study design. Besides cytokines, the vasodilatory agent NO is known to be overproduced during sepsis, contributing to hypotension and circulatory shock. We observed higher levels of vasodilatory NO_*x*_ in CLP animals treated with empty micelles compared to the SHAM group at 24 hr, but not compared to the CLP animals treated with TST-SSM.

TST-SSM also partially reduced renal damage but did not affect liver enzymes indicative of dysfunction during CLP-induced sepsis. A single injection of TST-SSM significantly decreased sepsis-associated plasma creatinine but not the levels of BUN. For the liver enzyme levels, ALT was unchanged among all groups, while AST was significantly higher in the septic mice treated with empty micelles compared to SHAM-control. TST-SSM treatment reduced the mean AST levels compared to the empty micelles, but due to the higher variability of the empty micelle group, the difference was not statistically significant. A previous report using a model of ischemic acute kidney injury showed that TLR9 activation did not impact renal function following renal ischemia but mediated secondary hepatic injury [29]. On the other hand, Yasuda et al. [8] demonstrated that chloroquine, a chemical inhibitor of TLR9, administered after 6 hr of CLP surgery reduced creatinine, BUN and ALT, but it had no significant effect on AST. However, in CLP-induced sepsis, whether TST-SSM protection is fully dependent on TLR9-mediated signaling remains unclear. Thus, further experiments comparing wild-type and TLR9 knockout mice would be important to address these results.

More importantly, TST-SSM treatment reduced the bacterial burden in the plasma and peritoneal cavity of septic mice. The gram staining of the bacteria present in those samples was not determined. TST typically targets Gram-positive bacteria due to the limited penetration through the Gram-negative outer membrane, but recent publication suggests that TST can be actively transported through pyoverdine receptors into Gram-negative bacteria such as *Pseudomonas aeruginosa* and *Actinobacter baumannii* under iron restricted conditions [30]. It is unknown if TST-SSM reduced bacterial burden by a direct antibacterial activity, indirectly by modulating the immune system, or both. Future experiments using alternative models of sepsis (LPS or *Escherichia coli* infection) can provide further support to understand the specific molecular mechanism behind the TST-SSM effect.

In this study, we confirmed the ability of sterically stabilized phospholipid micelles to deliver hydrophobic drugs such as TST *in vivo* for safe and efficacious therapeutic application. Drug encapsulation in PEGylated phospholipid micelles provides various benefits over other delivery systems, making it ideal for further clinical development [31]. SSM is a simple, reproducible nanocarrier composed of a single phospholipid ingredient (DSPE-PEG_2000_) with documented safety since it is present in the FDA-approved drug Doxil®. Recently, PEGylated phospholipids found an essential clinical application as they were developed into lipid nanoparticles to deliver Covid-19 mRNA vaccines by Pfizer/BionTech and Moderna [32, 33]. SSM enhances the solubility and stability of drugs by accommodating them in a hydrophobic core environment with a water-compatible PEGylated surface. This protective outer layer prevents particle opsonization and elimination by the reticuloendothelial system, thus enhancing the drug's biological half-life [34]. Due to its ideal nanosize, SSM targets loaded drugs to tissues with enhanced vascular permeability, characteristic of inflamed sites. In the case of sepsis, it is unknown the exact delivery site and site of action for TST and future studies are needed to explore the drug delivery mechanisms of SSM in sepsis. Finally, in our previous publication by Esparza and Onyuksel [19], we described a novel method of encapsulating TST using freeze-drying that is amenable to scaling up and can facilitate the translation of this nanomedicine to the clinics.

Although the primary effect of the TST-SSM nanomedicine is promising, as these research findings indicate a reduction in the inflammatory cytokines IL-6 and TNF-*α*, these cytokines represent only a portion of the broader inflammatory landscape. Markers such as CRP or leukocyte count were not evaluated in this study but could also provide valuable information on the effects of the TST-SSM nanomedicine. For future research, it would be beneficial to explore the specific mechanisms responsible for the anti-inflammatory effects of TST-SSM nanomedicine. For example, TST-SSM nanomedicine could be evaluated in septic TLR9^*−/−*^ mice to corroborate our findings. In addition, other models of sepsis could be used to understand the robustness of our findings and identify any other potential molecular targets of TST-SSM nanomedicine. TST has limited permeability through Gram-negative bacteria membranes; therefore, testing TST-SSM in a sepsis model induced by a known resistant Gram-negative bacteria could help us to clarify the contribution of the antibiotic effect of TST versus an anti-inflammatory response for the treatment of sepsis. Moreover, drug distribution studies upon iv administration could also be evaluated to better understand the effects of TST-SSM nanomedicine in sepsis. Currently, we hypothesize that TST-SSM acts at sites of inflammation where vacc9 activation would be high, including the peritoneum. However, a temporal analysis of tissue biopsies could provide insight into the accumulation of TST-SSM in inflamed tissues, shedding light on the pharmacokinetics of this novel treatment and its potential effectiveness. Previous drug distribution studies from our lab have shown that the SSM delivery system increased the accumulation of vasoactive intestinal peptide in the inflamed joints of arthritic mice compared to the free peptide drug [35]. The increased drug accumulation at the site of action was correlated with improved anti-inflammatory therapeutic effects and reduced toxicity (hypotension). The increased accumulation of SSM nanomedicines in inflamed tissues is attributed to the delivery system's ideal size (∼15 nm), which is large enough to keep it inside the normal blood circulation, but small enough to cross leaky vasculature characteristic of the inflamed tissues [36].

In conclusion, we successfully demonstrated the *in vivo* therapeutic use of injectable TST-SSM nanomedicine in a murine model of polymicrobial sepsis A single dose of TST-SSM nanomedicine (20 mg/kg) via intraperitoneal injection extended the median survival time of mice with CLP-induced sepsis from 31 to 44 hr compared to the empty micelles. Moreover, treated animals presented reduced plasma levels of pro-inflammatory cytokines IL-6 and TNF-*α*, partially improved renal function, and reduced bacterial burden in blood and peritoneal lavage fluid. Our findings confirmed the *in vivo* anti-inflammatory activity of TST-SSM nanomedicine, and if translated to the clinical setting, could prevent rapid death from septic shock and improve the chances of recovery by reducing the systemic pro-inflammation and bacterial burden.

## Data Availability

Data supporting this research article are available on reasonable request.
